# Electrical maturation of neurons derived from human embryonic stem cells

**DOI:** 10.12688/f1000research.4943.2

**Published:** 2014-10-01

**Authors:** Michael Telias, Menahem Segal, Dalit Ben-Yosef

**Affiliations:** 1 Wolfe PGD-SC Lab, Racine IVF Unit, Lis Maternity Hospital, Tel-Aviv Sourasky Medical Center, Tel-Aviv, 64239, Israel; 2 Department of Cell and Developmental Biology, Sackler Faculty of Medicine, Tel-Aviv University, Tel-Aviv, 64239, Israel; 3 Department of Neurobiology, Weizmann Institute of Science, Rehovot, 76100, Israel

## Abstract

In-vitro neuronal differentiation of human pluripotent stem cells has become a widely used tool in disease modeling and prospective regenerative medicine. Most studies evaluate neurons molecularly and only a handful of them use electrophysiological tools to directly indicate that these are genuine neurons. Therefore, the specific timing of development of intrinsic electrophysiological properties and synaptic capabilities remains poorly understood. Here we describe a systematic analysis of developing neurons derived in-vitro from human embryonic stem cells (hESCs). We show that hESCs differentiated in-vitro into early embryonic neurons, displaying basically mature morphological and electrical features as early as day 37. This early onset of action potential discharges suggests that first stages of neurogenesis in humans are already associated with electrical maturation. Spike frequency, amplitude, duration, threshold and after hyperpolarization were found to be the most predictive parameters for electrical maturity. Furthermore, we were able to detect spontaneous synaptic activity already at these early time-points, demonstrating that neuronal connectivity can develop concomitantly with the gradual process of electrical maturation. These results highlight the functional properties of hESCs in the process of their development into neurons. Moreover, our results provide practical tools for the direct measurement of functional maturity, which can be reproduced and implemented for stem cell research of neurogenesis in general, and neurodevelopmental disorders in particular.

## Introduction

*In-vitro* neural differentiation (IVND) of human pluripotent stem cells (hPSCs), is a promising vehicle for disease modeling and regenerative medicine ^1– 3^. Several protocols for IVND of hPSCs including embryonic stem cells (hESCs) or induced pluripotent stem cells (hiPSCs), are used worldwide, resulting in different neuronal types ^4, 5^. Upon IVND, confirmation of the neuronal fate is commonly analyzed by the expression of neuron-specific genes, including those of cytoskeletal components (e.g.; TUJ1, MAP2), transcription factors (e.g.; NeuN, NeuroD1) and synaptic proteins (e.g.; synaptotagmin, synaptophysin) ^6^. The expression of these genes indicates that the cell has acquired the machineries needed to build a neuron, but the ultimate indication that these are genuine neurons involves the analysis of their electrical properties. Most studies that do include electrophysiological recordings of hPSCs-derived neurons focus at one specific final time-point along the differentiation cascade, to demonstrate the neuronal identity that is related to the disease in study ^7– 9^. However, the analysis of the dynamics of electrical maturation at several time points can provide valuable insights into the pathology of neurodevelopmental disorders. Moreover, in the context of human embryonic neurogenesis, analyzing electrical maturation on hPSCs during IVND can shed light over molecular and cellular mechanisms that so far have been studied only using animal models.

Currently, only a few studies on IVND of hPSCs employed electrophysiological recordings at consecutive time points during neural differentiation, so far with inconsistent results, in terms of timing, frequency of action potentials and formation of spontaneous synaptic activity ^10– 13^. Although the timing of the development of neuronal electrical properties following IVND is extremely important, it currently remains poorly understood due to high variability in differentiation protocols, culture conditions and hPSC lines used. For this reason, the aim of this study was to systematically analyze, by electrophysiological tools, the developing neurons derived *in-vitro* from hESCs, in order to define predictive parameters for their electrical maturity, as well as to model the dynamics of neural development.

## Materials and methods

**Figure 1.**
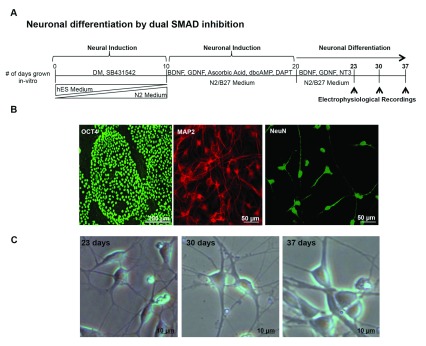
Neuronal differentiation of human embryonic stem cells by dual SMAD inhibition. ( **A**) Schematic representation of the protocol for *in-vitro* neural differentiation using dual SMAD inhibition. The protocol includes 3 steps: 1) Neural induction (days 0–10), by blocking SMAD signaling using Dorsomorphin (DM) and the TGF-β inhibitor SB431542 while reducing the relative amounts of hES medium and concomitantly increasing the relative amounts of N2 medium. 2) Neuronal induction (days 10–20), by incubating cells with brain-derived neurotrophic factor (BDNF), glia-derived neurotrophic factor (GDNF), ascorbic acid, dibutyryl-cyclic-AMP (dbcAMP) and the NOTCH1-inhibitor DAPT; 3) Neuronal differentiation (days 20–37), by dissociating cells and re-plating them on poly-lysine/laminin-coated glass coverslips in the presence of BDNF, GDNF and neurotrophin 3 (NT3). The specific time-points for electrophysiological recordings are indicated with arrows. ( **B**) Representative images of HUES-13 hESC line undifferentiated colonies stained positive for OCT4 (green); hESCs-derived neurons at day 21 stained positive for MAP2 (red) and hESCs-derived neurons at day 37 stained positive for NeuN (green). ( **C**) Bright field representative images of neurons at days 23, 30 and 37 of *in-vitro* neural differentiation.

**hESCs culture conditions and IVND:** The hESC line HUES-13 (kindly provided by the Melton Lab, Harvard University), was used in all experiments. hESCs were cultured on feeder layers of mitomycin C (Sigma)-inactivated mouse embryonic fibroblasts in hES-medium supplemented with bFGF (R&D), as previously described ^7^. Before induction of IVND, hESCs were cultured on Matrigel (BD)-coated wells for two passages. The dual SMAD inhibition IVND protocol was applied as previously described ^14^, including minor modifications. Figure 1A illustrates the actual IVND protocol used. Briefly, neural induction was achieved by gradually changing the medium from hES to N2 while adding dorsomorphin and SB431542 for 10 days; neuronal induction was achieved by changing the medium to N2/B27 and adding BDNF, GDNF, ascorbic acid, dbcAMP and DAPT for 10 additional days. At day 20 cells were dissociated using Accutase (Life Tech.) and seeded on 13 mm glass coverslips previously coated with 50 µg/ml Poly-D-Lysine and 20 µg/ml Laminin (Sigma). Seeding density was ~1.0×10 ^5^ cells/cm ^2^. From day 20 and on, neurons were continuously grown in N2/B27 medium supplemented with 20 ng/ml BDNF, GDNF and NT3. Concentrations of reagents and growth factors used were as follows: 5 µM dorsomorphin (Stemgent), 10 µM SB431542 (Stemgent), 20 ng/ml BDNF (PeproTech), 20 ng/ml GDNF (PeproTech), 0.2 mM ascorbic acid (Sigma), 0.5 mM dbcAMP (Sigma), 10 µM DAPT (Tocris), 20 ng/ml NT3 (PeproTech). N2 medium was composed of DMEM:F12 (Life Tech.), supplemented with 1% N2 (Life Tech.), 1% non-essential amino acids (BioInd.), 1% Glutamax (Life Tech.) and 100 µg/ml Primocin (InvivoGen). N2/B27 Medium was a 1:1 mixture of N2 and B27 media. B27 medium was composed of Neurobasal (Life Tech.), supplemented with 1% B27 (Life Tech.), 1% non-essential amino acids (BioInd.), 1% Glutamax (Life Tech.) and 100 µg/ml Primocin (InvivoGen).

**Cell patch-clamp:** Electrophysiological recordings were conducted as previously described ^7^. In brief, neurons on glass coverslips were transferred to a custom-made recording chamber adapted for an inverted microscope (Olympus XI-50), in standard recording medium, containing (in mM):10 HEPES, 4 KCl, 2 CaCl _2_, 1 MgCl _2_, 139 NaCl, 10 D-glucose (340 mOsm, pH 7.4). Cells were patch-clamped with glass pipettes (Sutter Instruments, 1.5 mm OD, 0.75 mm ID), pulled using a P87 Puller (Sutter Instruments). Pipettes contained intracellular medium composed of (in mM) 136 K-gluconate,10 KCl, 5 NaCl,10 HEPES, 0.1 EGTA, 0.3 Na-GTP, 1 Mg-ATP, and 5 phosphocreatine, pH 7.2 (pipette tip resistance was 5–8 MΩ). Action potentials were evoked (in current clamp mode) by injecting depolarizing current pulses. Membrane potential was held at -60 mV. Spontaneous synaptic currents were recorded in 2 minute sessions in voltage clamp mode with a 50 µs sampling rate. Signals were amplified with a Multiclamp 700B amplifier and recorded with Clampex9.2 software (Axon Instruments). Data were subjected to a 500-Hz low-pass filter and analyzed using Clampfit-9 and SigmaPlot.

**Immunofluorescence:** Immunostaining was performed as previously described ^7^. Briefly, cells were fixated for 15 minutes at R.T. using Cytofix (BD) and washed with PBS. Primary antibodies were applied at 4°C, overnight, in a PBS solution containing 2.5% BSA and 0.1% Triton. Staining with secondary antibody was performed for 1 hour at R.T., in the dark. The pluripotent gene Oct4 was detected with monoclonal mouse anti-human OCT4 (Santa Cruz, #sc-5279, RRID: AB_628051, Lot C1308, dilution – 1:200). Neurons were stained using polyclonal rabbit anti-human MAP2 (Santa Cruz, #sc-20172, RRID: AB_2250101, Lot D2710, dilution 1:250), and monoclonal mouse anti-human NeuN (GeneTex, #GTX30773, RRID: AB_1949456, Lot 27334, dilution 1:20). Primary antibodies were detected using sheep anti-mouse Cy2-conjugated and goat anti-rabbit Cy3-conjugated secondary antibodies (Jackson Labs).

**Imaging:** Bright field and fluorescence images of cells were obtained using an Olympus IX51 inverted light microscope, and a Zeiss LSM 700 confocal microscope. Images were processed using Olympus CellA for XP (2006) and ImageJ (NIH, v. 1.49) software.

**Statistical analysis:** Data were collected from 15–25 cells for each time point, in 2 different experiments. ANOVA was performed on data using SPSS (v. 19).

## Results

### Differentiation process and neuronal morphology

We have used a slightly modified version of an established protocol for IVND of hESCs ^5, 14^, that is based on the dual inhibition of the SMAD pathway ( Figure 1A). SMADs are the mammalian homologues to drosophila *mad* and C. elegans *sma*, and function as cytoplasmic mediators of TGFβ signaling ^15^. The process of IVND implemented here includes three major steps: (i) Neural induction by blocking SMAD signaling using Dorsomorphin and SB431542 ^16, 17^; (ii) Neuronal induction by incubating cells with pro-neuronal factors (BDNF, GDNF, ascorbic acid, dbcAMP) and the NOTCH1-inhibitor DAPT ^18^; (iii) Neuronal differentiation, by dissociating cells and re-plating them on poly-lysine/laminin-coated glass coverslips in the presence of BDNF, GDNF and NT3. Our hESCs selected for IVND were pluripotent as shown by the expression of OCT4 and the neurons derived were stained positive for MAP2 (>90% of cells), already at day 21 of IVND ( Figure 1B). At day 37, >90% of cells expressed the neuronal transcription factor NeuN ( Figure 1B). Typical neuronal morphology with 10–15 µm phase bright somata displaying pyramidal shape, extending an ‘apical’ dendrite and a few ‘basal’ dendrites was visible already at day 21 (MAP2 and NeuN in Figure 1B and Figure 1C). Importantly, the neurons did not undergo any significant morphological changes during later stages of differentiation ( Figure 1C, days 23, 30 and 37). Taken together, these results suggest that the cells analyzed during the three recording time-points ( Figure 1A) are probably early human embryonic neurons, which are practically impossible to study *in-vivo*.

### Time-dependent electrical maturation and firing of action potentials

**Figure 2.**
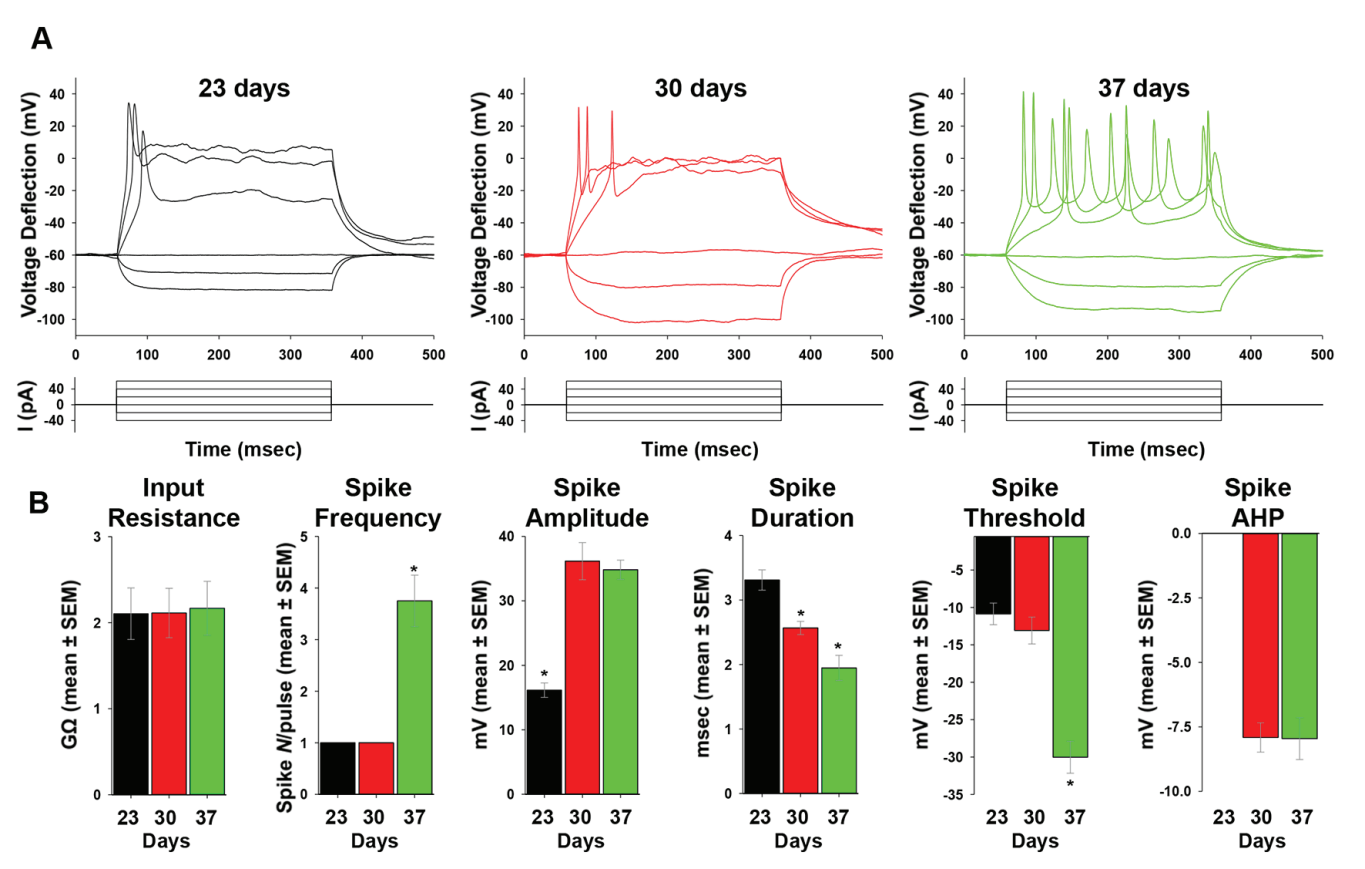
Current clamp recordings of hESCs-derived neurons. ( **A**) Representative traces for current clamp recordings at day 23 (black), 30 (red) and 37 (green). Membrane potential was held at ~-60 mV and voltage deflections (mV) are shown following 6 consecutive pulses of ~20 pA current injection from -40 to +60 pA. ( **B**) Data analysis showing (from left to right): input resistance (GΩ), spike frequency (spike number per pulse), spike amplitude (mV), spike duration (msec), spike threshold (mV) and spike after hyperpolarization (‘AHP’, mV), for the same recordings days as in ( **A**). Sample size: n=21 at day 23; n=25 at day 30; n=23 at day 37. Values are mean ± SEM. *P<0.05, ANOVA.

Current clamp recordings of neurons at days 23, 30 and 37 of IVND (corresponding to days 3, 10 and 17 days following induction of neuronal differentiation), showed a steady increase in the excitability of hESCs-derived neurons ( Figure 2A), while their input resistance remained similar for every time-point ( Figure 2B- **input resistance**). At days 23 and 30, these neurons could discharge only single action potentials (APs; 21 and 25 neurons were recorded at days 23 and 30 respectively). However, multiple spikes were observed in all neurons recorded at day 37 ( Figure 2A, **B-spike frequency**; 23 neurons were recorded). In addition, spike amplitude was significantly increased at day 30 and 37 as compared to day 23. Spike duration (measured at half-width of the action potential (AP), became shorter with prolonged differentiation, from 3.31±0.16 msec at day 23, to 2.57±0.10 msec at day 30, to a mean duration of 1.95±0.20 msec at 37 days ( Figure 2B). On the other hand, spike threshold was significantly lower for day 37 only. After hyperpolarization (AHP) potentials were not detected at day 23, but were present at day 30 and 37, in which the drop from spike threshold was ~-7.9 mV for both time-points. These results clearly show a steady ongoing process of electrical maturation for human *in-vitro* developing neurons, in which sequential firing of multiple spikes is achieved by day 37 following induction of IVND with the dual SMAD inhibition protocol. Furthermore, our results show that spike frequency, amplitude, duration, threshold and after hyperpolarization can serve as the best predictive measurements for electrical maturation.

### Time-dependent maturation of K ^+^ currents

**Figure 3.**
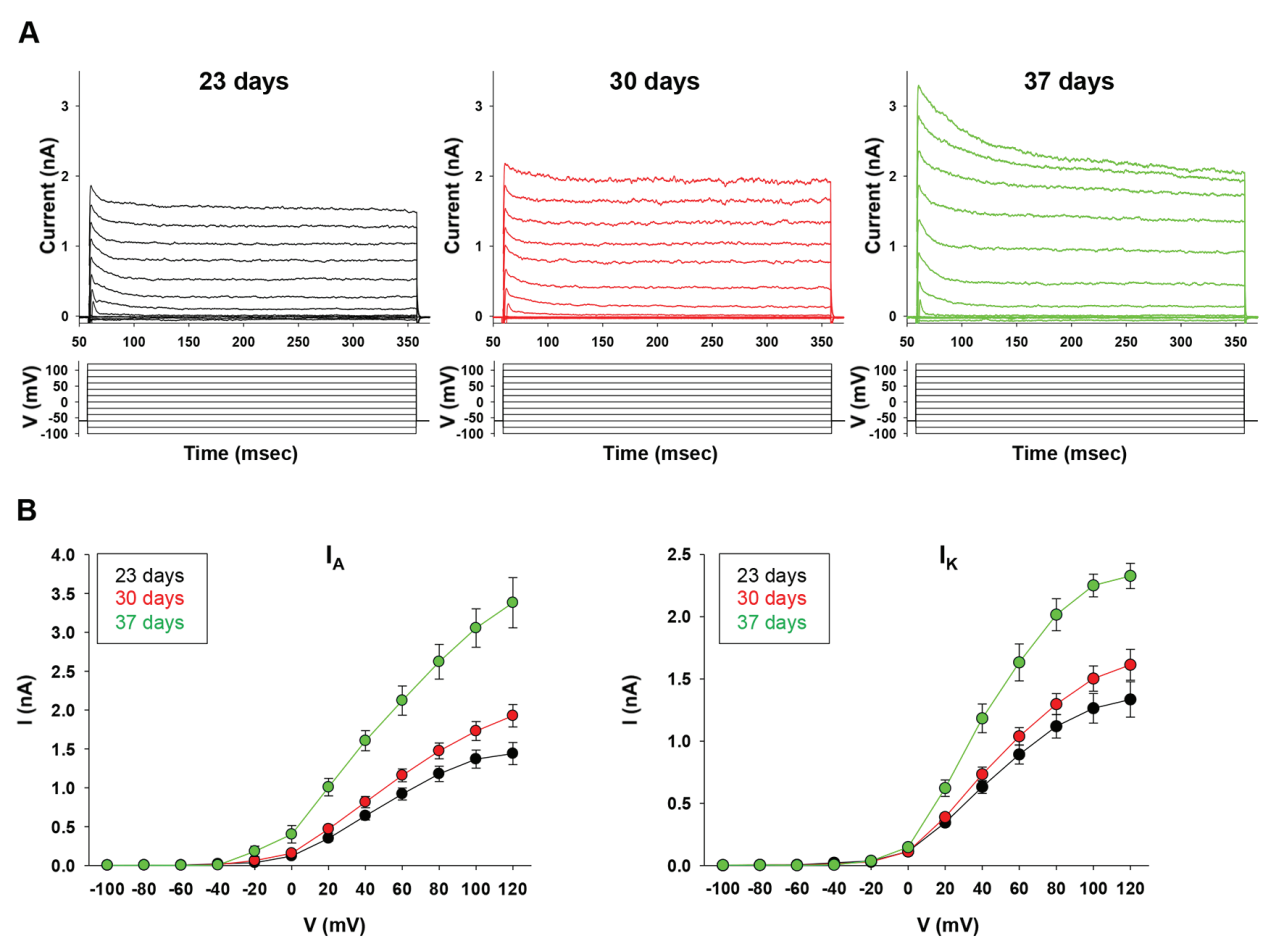
Voltage clamp recordings of K ^+^ currents. ( **A**) Representative traces for voltage clamp recordings at day 23 (black), 30 (red) and 37 (green). Membrane potential was held at -60 mV and current (nA) is shown following 12 consecutive pulses of 20 mV voltage steps from -100 to +60 mV. ( **B**) I-V curves for I _A_ and I _K_ currents for the same time-points as in ( **A**). Sample size: n=21 at day 23; n=25 at day 30; n=23 at day 37. Values are mean ± SEM.

In order to further explore the dynamics of time-dependent development of spike discharges, we examined properties of the derived neurons in voltage clamp mode at the same time-points listed above ( Figure 3A). We measured K ^+^ currents evoked by successive 20 mV voltage commands. The results show a significant difference between day 23 and 30 as compared to day 37 in the I-V curves of both the transient K ^+^ (I _A_) current and the sustained (I _K_) current ( Figure 3B; number of neurons recorded in each day was 21, 25 and 23 respectively). The sigmoid regression function that fits these curves is given by the equation: = $\frac{a}{1+e^{\frac{x–xo}{b}}}$, where ‘ *a*’ is the slope of the curve. Our calculations show that the slope of the curve in I _K_ increased from 1.36 (day 23), to 1.64 (day 30), and finally to 2.34 (day 37). Moreover, the value of *a* in I _A_ increased from 1.49 (day 23), to 2.01 (day 30), and finally to 3.50 (day 37). This robust increase in the slope of both K ^+^ currents from day 30 to day 37 is indicative that during this period the membrane of hESCs-derived neurons probably undergoes important changes in their expression of K ^+^ channels. In addition to the parameters we have measured in current-clamp for APs characteristics, the slope of I-V curves of K ^+^ currents could also be used as a tool to measure electrical maturity during IVND of hPSCs.

### Development of spontaneous synaptic activity

**Figure 4.**
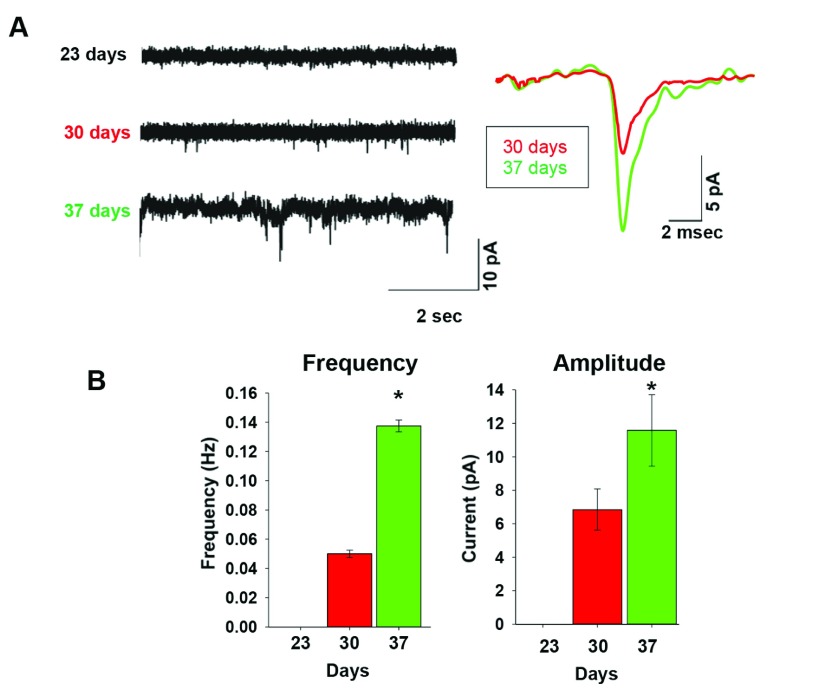
Voltage clamp recordings of spontaneous synaptic activity. ( **A**) Representative traces for spontaneous synaptic currents recorded in voltage clamp mode at -60 mV for days 23, 30 and 37. ( **B**) Analysis of spontaneous synaptic currents frequency (Hz) and amplitude (pA). Sample size: n=19 at day 23; n=18 at day 30; n=23 at day 37. Values are mean ± SEM. *P<0.05, ANOVA.

Finally, we examined the time-course of formation of spontaneous synaptic currents, indicating active synaptic connections ( Figure 4A). No synaptic activity was found in any tested cell at day 23 (n=19). However, ~50% of neurons at day 30 and 37 showed spontaneous synaptic activity, which increased from day 30 to day 37 in both frequency and current amplitudes (number of neurons recorded was 18 and 23 respectively) ( Figure 4B). No attempt was made to distinguish between excitatory and inhibitory synaptic currents in the present study. At day 37, spontaneous synaptic currents had a mean rise time of 1.84±0.16 msec, and their mean decay time was 3.25±0.71 msec. These results suggest that the capability of *in-vitro* hESCs-derived neurons to develop synaptic connections can arise at a relatively early time-point during IVND, and is concomitant with the developmental timing of burst firing. Furthermore, these results indicate that electrical maturation involves the development of intrinsic properties in individual neurons, in parallel with the development of network-activity.

Data files Electrical maturation of neurons derived from human embryonic stem cells Dataset 1:  Additional images of raw microscopy showing neurons derived from hESCs in bright field and stained, as well as original images for a single hESC colony (corresponding to Figure 1B-C).
Dataset 2: Data are divided as following: - I-V protocol tested in current clamp modality (“Current Clamp I-V”), corresponding to results shown in Figure 2. - I-V protocol tested in voltage clamp modality (“Voltage Clamp I-V), corresponding to results shown in Figure 3. - Recordings of spontaneous activity at -60 mV (“Voltage Clamp spontaneous”), corresponding to results shown in Figure 4.

## Discussion

Currently, there is no standard protocol to analyze the developmental stage in which all the required electrophysiological properties for proper function are already present in neurons derived by IVND of hPSCs. Several studies provide electrophysiological data on hPSCs-derived neurons but they do so only for a single specific end-point of the process, in order to compare control neurons to diseased ones ^7– 9, 19– 26^. However, the dynamic of electrical maturation at several time-points along the process has not been extensively investigated, and currently there are only a few studies which addressed this question, with inconsistent results ^10– 13, 27– 29^. Here, we have analyzed systematically the electrical maturation of human neurons derived from hESCs. We show that, by applying the dual SMAD inhibition in the IVND protocol on hESCs, early embryonic neurons are generated demonstrating electrical maturation already by day 37, including firing of spike bursts with increased amplitude and reduced duration. Our results further show that this electrical maturation is accompanied by a steady increase in K ^+^ currents, which enabled faster and more reliable repolarization. Moreover, a steady and gradual increase in spontaneous synaptic activity is observed at the same three time-points, suggesting that electrical maturation occurs not in individual neuron, but also in the developing neuronal networks. Nevertheless, no spontaneous action potential discharges could be detected, indicating that the network is still not fully functional.

As shown here, the neurons we derived could fire trains of APs not before day 37 (~5 weeks) of IVND. Indeed, a study in which the same dual SMAD inhibition protocol was used to generate neurons, spike bursts were measured already by week 4, but this was observed only in ~40% of the hESCs-derived neurons ^12^, as compared to our results demonstrating spike bursts in 100% of the neurons at the same time. In comparison, other studies have shown that when dopaminergic neurons or GABAergic interneurons were derived from hPSCs, a similar phenomenon of time-dependent electrical maturation was observed, but only following >8 weeks of IVND ^11, 13, 28^. Furthermore, other IVND protocols applied on hESCs, showed no incidence of burst firing and no significant differences between time-points in APs parameters ^10, 27^. Our results indicate that measurement of spike frequency, amplitude, duration, threshold and after hyperpolarization can serve as predictive parameters for electrical maturity. Interestingly, spike duration was found to be the most reliable predictor parameter, and its measurement at each time-point tested. Similar to APs, K ^+^ currents reflect the process of electrical maturation in a time-dependent manner. In addition, we have established that the slope of I _K_ and I _A_ steadily increases with time. Indeed, in the study of Takazawa *et al*., 2012, hESCs-derived spinal motor neurons demonstrating bursts of multiple APs at day 36, also showed a time-dependent maturation in the transient and in the sustained K ^+^ currents (I _A_ and I _K_, respectively, ^29^). Furthermore, when neural differentiation through dual SMAD inhibition was performed to produce forebrain neurons, as we have done in this study, a time-dependent increase in the amplitude of both I _A_ and I _K_ was indeed shown during the first 4 weeks ^12^. In contrast, Nicholas *et al*., 2013 showed in hPSCs-derived GABAergic interneurons a steady increase of an unspecified K ^+^ current, reaching a maximal peak of ~1.5 nA only after 30 weeks of IVND ^13^, but Hartfield *et al*., 2014 showed no significant changes between relevant time-points in the average peak amplitude of K ^+^ currents in hPSCs-derived dopaminergic neurons ^27^. These observations indicate that development of K ^+^ currents takes place during the earliest stages of electrical maturation. However, more research is needed to understand how and when these K ^+^ channels are expressed on the membranes of *in-vitro* developing neurons, and whether the increase in their current is caused by an increase in their density throughout the membrane or by maturation of their intrinsic activation properties.

We have shown here time-dependent development of spontaneous synaptic activity, at a relatively early time-point during IVND, and concomitant with the developmental timing of burst firing, indicating that electrical maturation involves the development of intrinsic properties in individual neurons, in parallel with the development of network-activity. Other studies however, have shown that although action potential can be produced at earlier stages of differentiation, the generation of neuronal networks as evidenced by spontaneous synaptic activity, is observed only at later stages of IVND, (at least 8 weeks of differentiation into GABAergic forebrain interneurons ^13, 28^, or 10 weeks of differentiation into dopaminergic midbrain neurons ^11^). It has been suggested that IVND of hESCs produces immature embryonic-like neuronal cells, which take several months to develop the characteristic genetic and electrophysiological properties of mature adult-like neurons ^30, 31^. Others have proposed that IVND of hPSCs mimic the real time-frame of *in-vivo* human embryonic neurogenesis, due to an “intrinsic clock-like mechanisms” ^32^. It is commonly accepted that synaptogenesis in humans starts only by the end of fetal life and during the first months of postnatal life ^6, 33^. Nevertheless, here we show significant spontaneous synaptic activity already by day 37, suggesting that *in-vitro* conditions results in an accelerated rate of maturation and development. The different timing of neuronal functional maturation observed in the different studies could be explained by high variability in cultures due to the different hPSCs used, different IVND protocols, different seeding density of cells, and other factors related to the IVND protocol used.

In conclusion, our results shed light on the dynamic development of the electrophysiological properties of individual neurons as well as *in-vitro* neuronal networks. Furthermore, these findings suggest critical electrophysiological parameters that can be used to predict the precise timing in which neuronal functionality is acquired by human cells developing *in-vitro*. Therefore, the results of the present study provide a valuable tool for the direct measurement of electrical maturity, which can be implemented when studying neurodevelopmental and neurodegenerative diseases. Timing of electrical maturation can greatly vary among different cell lines of hESCs and hiPSCs, as well as between different protocols for IVND. Therefore, analysis of the parameters proposed in this study, which are universal for neuronal electrical activity and easy to reproduce, could serve to calibrate and adjust the time-course of electrical maturation in every cell line and for every protocol.

## Data availability

figshare: Data files electrical maturation of neurons derived from human embryonic stem cells doi: 10.6084/m9.figshare.1132475 ^34^
