# Supplementary material for: Electrical maturation of neurons derived from human embryonic stem cells
Source: F1000Res. 2014 Oct 1;3:196. Originally published 2014 Aug 19. [Version 2] doi: 10.12688/f1000research.4943.2 (PMC4184377; doi:10.12688/f1000research.4943.2)
Supplement: Peer review history

# Peer review: Referee response for version 1

**DOI:** 10.5256/f1000research.5279.r5862

**Published:** 2014-09-24

**Authors:** Shauna Yuan

**Affiliations:** 1 Department of Neuroscience, University of California San Diego, La Jolla, USA

This is an interesting article examining the time course of the electrophysiological properties of neurons derived from human embryonic stem cells.  The authors studied firing of action potentials, potassium currents and spontaneous synaptic activities.  They found that all of these properties gradually become more mature over time.  This study shows the feasibility of conducting such experiments to map out developmental stages of neurons in a culture dish.  The title and abstract convey the message of the manuscript.  It is well-written and contains comprehensive information on the study design, methods and analysis.  The data is well presented.  It is not clear from the article that the three properties that the authors looked at are fully developed at 37 days.  Can they comment on the variability between cultures?  In the conclusion, the authors could possibly expand on what they think about the timing of maturation.  Would the days of maturation be very different due to the differentiation method; therefore, such time-course should be performed for each new differentiation protocol?

I have read this submission. I believe that I have an appropriate level of expertise to confirm that it is of an acceptable scientific standard.

# Response

**Published:** 2014-09-28

**Authors:** Michael Telias

The authors wish to thank the referee for her kind words and the time to review our work.

Specific comments:

1. *“It is not clear from the article that the three properties that the authors looked at are fully developed at 37 days. Can they comment on the variability between cultures?”  *
  The points raised by the referee are indeed interesting. Variability among cultures (different experiments with the same hESC line) was relatively low, as revealed by the standard error of the mean in the parameters measured, such as spike amplitude, duration, etc. (see Fig. 2B).
  Regarding the capability of cells of firing consecutive trains of action potentials: in this study we have recorded neurons up to day 37 following IVND, and concluded that these 37 days old neurons are already functionally mature. A continuous study, that we are currently conducting at our lab, demonstrates that in >60 days old neurons the only parameter that change, as compared to 37-days old neurons, is spike frequency but all other parameters remain the same (i.e., spike amplitude, duration, threshold, AHP).
2. *“In the conclusion, the authors could possibly expand on what they think about the timing of maturation.  Would the days of maturation be very different due to the differentiation method; therefore, such time-course should be performed for each new differentiation protocol?”*
  According to the referee’s comment, we have added the following explanation to the conclusion (last paragraph of Discussion): “Timing of electrical maturation can greatly vary among different cell lines of hESCs and hiPSCs, as well as between different protocols for IVND. Therefore, analysis of the parameters proposed in this study, which are universal for neuronal electrical activity and easy to reproduce, could serve to calibrate and adjust the time-course of electrical maturation in every cell line and for every protocol”

# Peer review: Referee response for version 1

**DOI:** 10.5256/f1000research.5279.r5864

**Published:** 2014-09-01

**Authors:** Maija Castren

**Affiliations:** 1 Institute of Biomedicine, University of Helsinki, Helsinki, Finland

Telias *et al.* report a systematic analysis of intrinsic electrical properties of developing neurons differentiated from pluripotent human embryonic stem cells during neuronal maturation days 23-37 *in vitro*. Neuronal differentiation was performed by dual SMAD inhibition and differentiated neurons showed mature neuronal morhology. Current clamp recordings showed changes in spike amplitude, frequency and duration and sequential firing of multiple spikes was found by day 37. At the same time a significant increase in the frequency and amplitude of spontaneus synaptic activity could be detected and an augmentation of K ^+^ currents. The provides new information about the dynamic development of the electrophysiological properties of neurons differentiated from pluripotent cells.

The manuscript is well written and discussed. The title is appropriate for the paper. The methods are sound. 

The following points need to be addressed:

- Page 3, the second paragraph; it is not clear how images are indicated (Figure 1B-MAP2, Figure 1C).
- The figure 4B; Is the difference of frequency between values at day 30 and 37 significant with *? The presentation gives an impression that the difference is more significant.

I have read this submission. I believe that I have an appropriate level of expertise to confirm that it is of an acceptable scientific standard.

# Response

**Published:** 2014-09-28

**Authors:** Michael Telias

The authors wish to thank the referee for her kind words and the time to review our work.

Specific comments:

- *"Page 3, the second paragraph; it is not clear how images are indicated (Figure 1B-MAP2, Figure 1C).”*
  We have revised the text in the Results section “Differentiation process and neuronal morphology” to correct this mistake, as explained by the referee, and make the text more comprehensible: “[…] (MAP2 and NeuN in Figure1B and Figure 1C).”
- "The figure 4B; Is the difference of frequency between values at day 30 and 37 significant with *? The presentation gives an impression that the difference is more significant."
  Indeed the p value in the frequency graph is <0.01, as noted by the referee. However, we chose to symbolize every p value that is below 0.05 with only one asterisk, regardless of its specific value.
